# Multi-omics analysis of pigmentation related to proanthocyanidin biosynthesis in brown cotton (*Gossypium hirsutum* L.)

**DOI:** 10.3389/fpls.2024.1372232

**Published:** 2024-03-13

**Authors:** Doug J. Hinchliffe, Marina Naoumkina, Gregory N. Thyssen, Sunghyun Nam, SeChin Chang, Jack C. McCarty, Johnie N. Jenkins

**Affiliations:** ^1^ Cotton Fiber Bioscience and Utilization Research Unit, United States Department of Agriculture (USDA), Agricultural Research Service (ARS), New Orleans, LA, United States; ^2^ Cotton Quality and Innovation Research Unit, USDA-ARS, New Orleans, LA, United States; ^3^ Genetics and Sustainable Agriculture Research Unit, USDA-ARS, Mississippi State, MS, United States

**Keywords:** brown cotton, proanthocyanidins, metabolites, mass spectrometry, *Gossypium hirsutum*, flame-retardant

## Abstract

Naturally-colored brown cotton (NBC) fiber is an environmentally friendly raw source of fiber for textile applications. The fiber of some NBC cultivars exhibits flame-retardant properties, which can be used in textiles that require flame resistance. Proanthocyanidins or their derivatives are responsible for the brown pigment in NBC; however, how flame retardancy is related to pigmentation in NBC is poorly understood. To gain insight into brown pigment biosynthesis, we conducted comparative transcripts and metabolites profiling analysis of developing cotton fibers between the brown (MC-BL) and white (MC-WL) cotton near-isogenic lines (NILs), genetically different only in the *Lc1* locus. In this study, mass spectrometry was used to detect metabolites in BL and WL developing fibers at 8, 12, 16, 20, 24, 36, and 40 days post anthesis (DPA) and mature fibers. Transcripts analysis was performed at two critical fiber developmental points, 8 DPA (fiber elongation) and 20 DPA (secondary cell wall deposition). We found 5836 (ESI MS positive mode) and 4541 (ESI MS negative mode) metabolites significantly different accumulated between BL and WL. Among them, 142 were known non-redundant metabolites, including organic acids, amino acids, and derivatives of the phenylpropanoid pathway. Transcript analysis determined 1691 (8 DPA) and 5073 (20 DPA) differentially expressed genes (DEGs) between BL and WL, with the majority of DEGs down-regulated at 20 DPA. Organic acids of the citric acid cycle were induced, while most of the detected amino acids were reduced in the MC-BL line. Both *cis*- and *trans*-stereoisomers of flavan-3-ols were detected in developing MC-WL and MC-BL fibers; however, the gallocatechin and catechin accumulated multiple times higher. Gas chromatography-mass spectrometry (GC-MS) analysis of fatty acids determined that palmitic acid long-chain alcohols were the main constituents of waxes of mature fibers. Energy-dispersive X-ray spectrometry (EDS) analysis of mature fibers revealed that potassium accumulated three times greater in MC-BL than in MC-WL mature fibers. This study provides novel insights into the biosynthesis of pigments and its association with flame retardancy in NBC fibers.

## 1 Introduction

Environmentally friendly fibers of naturally-colored brown cotton (NBC) varieties are most commonly used for fabrics. Due to pigments, clothes from NBC fibers resist fading colors during laundry and protect the skin from ultraviolet radiation. Fibers of some NBC varieties exhibited flame retardant (FR) properties that can offer an eco-friendly alternative to synthetic FR additives in textiles.

Pigments responsible for the color of NBC are proanthocyanidins (PAs), also called condensed tannins. PAs are polymeric flavan-3-ols whose chemical structure consists of benzopyran (A and C rings) linked with another aromatic ring (B ring) at the C2 position (Yu et al., 2023). The common PA subunits are catechin and epicatechin with the gallate modification of the hydroxyl group at the C3’ position on the B-ring (Dixon et al., 2005; Xie and Dixon, 2005). The difference between catechin and epicatechin is the stereochemistry around the 2 and 3 positions of the C-ring; catechin is 2,3-*trans*-2R,3S-flavan-3-ol, while epicatechin is 2,3-*cis*-2R,3R-flavan-3-ol. Flavan-3-ols are synthesized from leucoanthocyanidins or anthocyanidins through the flavonoid pathway. Catechin is produced from leucoanthocyanidin by removing the hydroxyl group at the C4 position catalyzed by the leucoanthocyanidin reductase (LAR). Epicatechin is converted from anthocyanidins by anthocyanidin reductase (ANR) (Xie et al., 2003; Zhu et al., 2015).

Initially, PAs were detected in NBC fibers with DMACA (*p*-Dimethylaminocinnamaldehyde) staining (Xiao et al., 2007; Li et al., 2012). A small amount of PAs was also detected in early-developing white fibers, which gradually disappeared at the later stage of development (Li et al., 2012). Both isoforms of PA subunits, catechin and epicatechin, were detected in NBC by using liquid-chromatography mass spectrometry (LC-MS), nuclear magnetic resonance (NMR), and a matrix-assisted laser desorption/ionization-time of flight mass spectrometry (MALDI-TOF MS). Using LC-MS analyses, one study detected catechin and gallocatechin as the most abundant PAs in NBC fibers (Xiao et al., 2014). Another study determined by NMR and MALDI-TOF MS analyses that epicatechin and epigallocatechin were the most predominant PAs in NBC fibers (Feng et al., 2014). The dimeric PAs, procyanidin (PC) and prodelphidin (PD) were detected in white and NBC fibers; however, their ratios were different: the amount of PC and PD was equal in white fibers, whereas NBC fibers contained mainly PD units (Feng et al., 2014). The oxidation products of PAs, quinones, are responsible for color development in NBC fiber since developing fibers do not show distinct coloration until maturation (Feng et al., 2014).

Transcription factors, including TT2 (MYB123), TT8 (bHLH42), and TTG1 (WD40 family), are primary regulators of PA biosynthesis (Yu et al., 2023). In cotton, at least six incompletely dominant genetic loci (*Lc1* – *Lc6*) have been associated with brown fiber (Kohel, 1985). The *Lc1* was mapped to a 1.4 Mb inversion on chromosome A07 upstream of a TT2 homologous gene (*Gh_A07G2341*) (Hinchliffe et al., 2016). An independent mapping study determined that two QTLs in the *Lc1* locus regulate the shades of brown color in fibers. The *qBF-A07-1* (*Gh_A07G2341* - TT2) contributes to the brown color, while the *qBF-A07-1* and *qBF-A07-2* (*Gh_A07G0100* - TTG1) regulate the shades of brown fiber (Wen et al., 2018). A recent study demonstrated that the *Gh_A07G2341 (GhTT2-3A)* overexpression in cotton activated PA structural genes and PA biosynthesis that resulted in brown mature fiber in transgenic lines (Yan et al., 2018).

The source of FR in the NBC remains unclear and is not entirely correlated with the intensity of color in different NBC cultivars (Hinchliffe et al., 2015). One study suggested that higher FR in NBC is linked to condensed tannins and sodium content; authors demonstrated sequestration of inorganic salts such as sodium through ionic interactions with the partial negative charges generated by adjacent hydroxyl units on the B-ring of flavonoid units in PAs and the possible formation of flavonoid–metal complexes in NBC (Nam et al., 2016). Another study argued that condensed tannin is not the source of FR in brown cotton since enhanced FR and anthocyanin precursors appear in developing fibers well before the brown color is detectable; authors suggested that the unknown FR component is probably sequestered by PAs or PA precursors via metal-flavonoid complexes (Hinchliffe et al., 2016).

The current study aims to understand the relationship between pigmentation and FR in NBC. To gain insight into PA biosynthesis in NBC, we conducted comparative metabolite profiling analyses of developing cotton fibers between the brown (MC-BL) and white (MC-WL) near-isogenic lines (NILs). We found significant changes in metabolite accumulation not only in secondary but in primary metabolism as well. Both isoforms of PA subunits, (+)-catechin and (-)-epicatechin, were detected in NBC and white fibers, with the relative amount of catechin greater than epicatechin. Gas chromatography-mass spectrometry (GC-MS) analysis of fatty acids determined that palmitic acid and long-chain alcohols, 1-Octacosanol and 1-Triacontanol, were predominant in cotton fiber waxes. Scanning electron microscopy and elemental mapping analysis of mature fibers revealed potassium accumulation three times greater in NBC than in white fibers.

## 2 Materials and methods

### 2.1 Plant materials

A spontaneous mutation that changed fiber color from white to brown on one branch of the same plant was observed in a cotton (*G. hirsutum* L.) line of unknown background in Starkville, MS (Hinchliffe et al., 2016). The seeds from white and brown cotton bolls from this single plant were collected and designated as MC-WL and MC-BL. The lines differ in *Lc1* locus only; therefore, they are near-isogenic lines (NILs). Both lines continued to maintain their corresponding fiber color through the following generations. The lines were further advanced by self-pollination single seed descent for three generations in New Orleans, LA. Pure lines of MC-WL and MC-BL were planted in the field in New Orleans, LA, in 2017 and grown using standard agricultural practices. Seeds for each cotton line were planted in rows measuring 20 m x 1 m with approximately 50 plants per row. Three rows were planted for each line, with each row used as a biological replicate for sample harvest. Developing cotton fibers were collected from the MC-WL and MC-BL cotton lines at 8, 12, 16, 20, 24, 32, 40 DPA, and mature fibers from open bolls. Mature seed cotton was hand-harvested and ginned using a laboratory roller gin.

### 2.2 Ultra high performance liquid chromatography-mass spectrometry analysis

Developing (8, 12, 16, 20, 24, 32, and 40 DPA) and mature (~50 DPA) cotton fibers in three biological replicates from MC-WL and MC-BL were used for metabolomic analysis. Fiber samples (15 mg each) were extracted using 8:1:1 acetonitrile: methanol: acetone ratio. Southeast Center for Integrated Metabolomics University of Florida performed sample extraction and metabolome analysis. Global metabolomics profiling was performed on a Thermo Q-Exactive Orbitrap mass spectrometer with Dionex UHPLC and autosampler. Catechins standards were run along with the samples to confirm their presence in the samples. All samples were analyzed in positive and negative heated electrospray ionization with a mass resolution of 35,000 at m/z 200 as separate injections. Separation was achieved on an ACE 18-pfp 100 x 2.1 mm, 2 μM column with mobile phase A as 0.1% formic acid in water and mobile phase B as acetonitrile. The flow rate was 350 μL/min with a column temperature of 25°C. Aliquot of 4 μL was injected for negative ions and 2 μL for positive ions.

### 2.3 GC-MS analysis of fatty acids

Long chain alcohol standards, 1-Octacosanol and 1-Triacontanol, and Supelco 37-component FAME mix (C4 – C24) were obtained from Sigma-Aldrich (St. Louis, MO); MSTFA+1% TMCS was purchased from Thermo Scientific (Bellefonte, PA). Cotton waxes were extracted from 1 g of mature fibers with dichloromethane using an Dionex ASE 350 Accelerated Solvent Extractor (Thermo Fisher Scientific, (Waltham, MA). Extracts were taken to dryness under a nitrogen stream.

Esterification of fatty acids was performed as follows: 1 mg of dry extract was dissolved in 1 ml of diethyl ether and mixed with 20 µl of methyl acetate and 40 µl of 0.5M sodium methoxide in anhydrous methanol and incubated for 5 min; 30 µl of saturated oxalic acid in ether was added to precipitate glycerides; samples were centrifuged for 7 min and supernatants were transferred to new glass vials, dried in SpeedVac and diluted in 200 µl hexane; injection volume was 1 µl.

Derivatization of long chain alcohols was performed as follows: 10 µl of MSTFA + 1% TMCS were added to 1 mg of dry extracts; incubated at 50°C for 1 hour; 200 ul of hexane was added, mixed, and transferred into a vial for GC automatic sampling; injection volume was 100 split of 1 µl.

The fatty acids and long chain alcohols were analyzed by GC-MS using an Agilent 6890 GC, 5973 MS, and Gerstel MACH (Agilent LTM) fast GC adaptation, using an HP-88 LTM column (30 m × 0.18 mm, 0.18 μM; Agilent). The column heat gradient was 50°C (1 min hold) to 140°C at 25°C/min, followed by a 4°C/min gradient to 177°C and a 2°C/min gradient to 210°C (1.65 min hold). A pressure ramp program was set up as 29.2 psi (1 min hold) to 39.3 at 2.81 psi/min, followed by a 0.44 psi/min gradient to 43.4 psi and a 0.22 psi/min gradient to 47.1 psi (1.65 min hold), which kept a 1 mL/min helium flow rate. The inlet was set to a 50:1 split at 220°C, using an insert with glass wool (Agilent 5062-3587). The GC oven and MS transfer were set at 250°C. A 5 μL syringe was used in a 7683 autosampler. A mass range of m/z 35−500 was acquired.

Concentrations of wax components were determined using standard curves of commercially available FAMEs and long chain alcohols; the peak areas of target ions were used for concentration calculation; the peak areas were normalized by the area of the internal standard C19:0 for a specific sample. Analysis was performed in five technical replicates from two independent isolations of waxes from mature cotton fibers.

### 2.4 RNA isolation and RNAseq

Total RNA was extracted using the Sigma Spectrum Plant Total RNA Kit (Sigma-Aldrich, St Louis, MO, USA) with on-column DNase I digestion following the manufacturer’s protocol. RNA quantity and integrity were evaluated as previously described (Hinchliffe et al., 2011).

Total RNA from 8 and 20 DPA developing fibers of MC-BL and MC-WL in two biological replicates were used for library preparation and RNAseq (LC Sciences, Houston, TX, USA). Sample preparation and library constructions were performed using TruSeq Stranded mRNA Library Prep Kit (Illumina Inc., San Diego, CA, USA) following the manufacturer’s protocols. Samples were sequenced using a HiSeq 2000 (Illumina Inc.) with 100 bp paired-end reads. Raw sequence reads were filtered for quality and trimmed by SICKLE (Joshi and Fass, 2011) and aligned to the draft *G. hirsutum* TM-1 reference genome (Zhang et al., 2015) with GSNAP software (Wu and Nacu, 2010). Reads mapping to annotated genes were counted using BEDTools software (Quinlan and Hall, 2010).

### 2.5 Energy-dispersive X-ray spectroscopy

EDS and elemental mapping analyses of fiber surfaces in scanning electron micrographs (SEM) were conducted with an accelerating voltage of 15 kV using a Phenom G6 ProX SEM (Nanoscience Instruments, Phoenix, AZ, USA) equipped with an EDS detector. The sample was mounted on a stub using double-sided carbon tape, and then a seven nm-thick gold coating was placed onto the sample using a LUXOR gold sputter coater (Aptco Technologies, Nazareth, Belgium).

### 2.6 Statistical analyses

Statistical analyses of UHPLC-MS and RNAseq data of cotton fiber samples were performed using JMP Genomics 10 software (SAS Institute Inc. Cary, NC, USA). An ANOVA was conducted to find differentially accumulated metabolites or expressed genes between MC-BL and MC-WL as previously described (Naoumkina et al., 2013, 2014). The metabolites or genes that accumulated or expressed differently between two lines by at least 2-fold with a false discovery rate < 0.05 (Benjamini and Yekutieli, 2001) were considered significant. A two-way ANOVA of fatty acids was conducted with Prism 9 (GraphPad Software, Inc.).

## 3 Results

### 3.1 Global metabolite changes in NBC fibers

**Figure 1:**
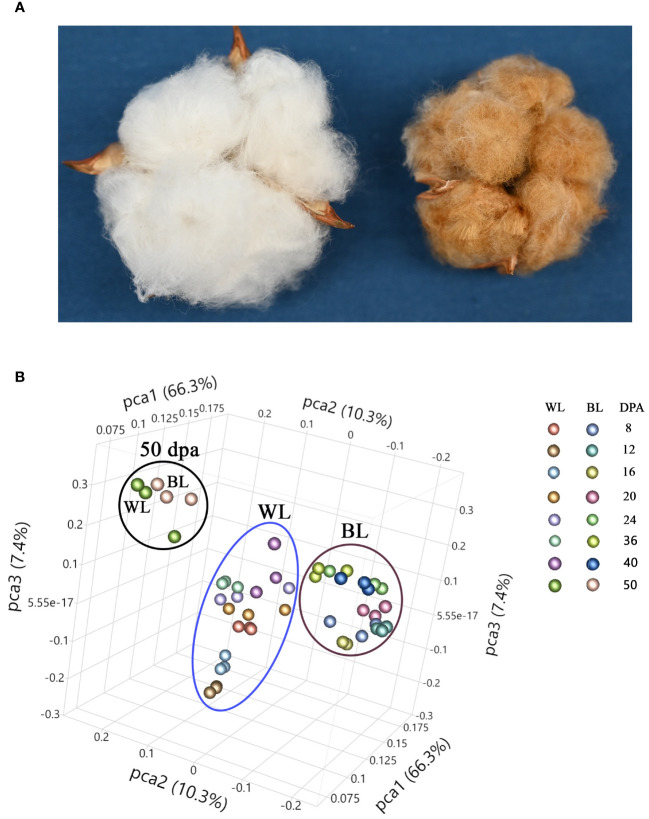
Phenotype and principal component analysis (PCA) of white and NBC fiber samples. **(A)** Images of open bolls of MC-WL and MC-BL NILs. **(B)** PCA of fiber samples based on UHPLC-MS metabolome data. WL, white line MC-WL fiber samples, and BL, brown line MC-BL fiber samples. Each fiber developmental time-point is represented by three biological replicates (annotation on the right).

**Figure 1A** shows images of open bolls of MC-WL and MC-BL near-isogenic lines (NILs). The mature fiber length of MC-BL was about 14 mm, whereas MC-WL fiber was about 27 mm. Developing (8, 12, 16, 20, 24, 32, and 40 DPA) and mature (50 DPA) fibers of white (MC-WL) and brown (MC-BL) cotton were used for metabolite analysis. Each sample (in three biological replicates) was analyzed in positive and negative heated electrospray ionization on a Thermo Q-Exactive Orbitrap mass spectrometer. A total number of detected peaks, 9140 (positive) and 5895 (negative), were used for principal component analysis (PCA) to explore the relationship between brown and white fiber samples in metabolite pools (**Figure 1B**). According to PCA, samples from developing fibers (8, 12, 16, 20, 24, 32, and 40 DPA) separated between white and brown cotton, while samples from mature fibers formed a distinct cluster (**Figure 1B**). Therefore, a substantial difference in metabolite composition (in the detectable range of molecular weights by the UHPLC-MS) was observed between MC-WL and MC-BL in developing fibers.

**Figure 2:**
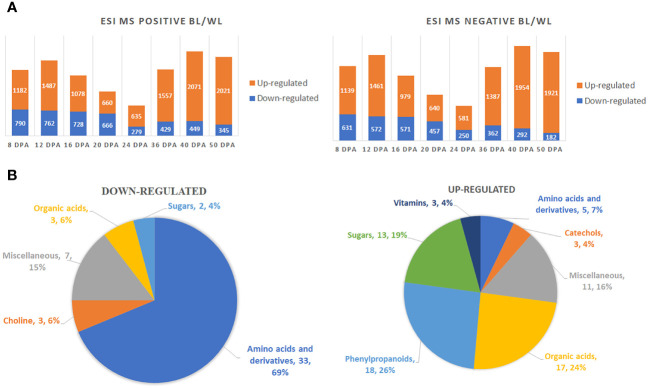
Global metabolite changes in developing fibers of brown vs. white cotton lines. **(A)** Bar charts of up-regulated and down-regulated metabolites in brown fibers according to ESI MS positive and negative detection modes. Digits on the bars represent the number of detected metabolites by two detection modes, significantly accumulated at least 2-fold differently between brown and white cotton lines. Fiber development time points are 8, 12, 16, 20, 24, 36, 40 and 50 DPA. BL, brown cotton line MC-BL, and WL, white cotton line MC-WL. **(B)** Pie chart of down-regulated and up-regulated metabolites in brown fibers. Quantity and percentage of metabolites are shown in each category.

A two-way ANOVA determined that 5836 (ESI MS positive mode) and 4541 (ESI MS negative mode) metabolites were significantly different in accumulation between brown and white fibers (**Supplementary Data 1**). **Figure 2A** shows the distribution of up-regulated and down-regulated compounds in MC-BL vs. MC-WL developing and mature fiber samples. The highest number of peaks was detected by both methods at 40 DPA and the lowest at 24 DPA of fiber development. There were significantly more up-regulated than down-regulated metabolites at each developmental time point.

Among detected peaks, 142 were non-redundant known metabolites, 70 of which were significantly (FDR < 0.05) up-regulated and 48 down-regulated (**Supplementary Tables 1, 2**). Amino acids and their derivatives were the main category among down-regulated metabolites, whereas phenylpropanoids, organic acids, and sugars were the major categories among up-regulated metabolites (**Figure 2B**).

### 3.2 Global transcript changes in NBC fibers

**Figure 3:**
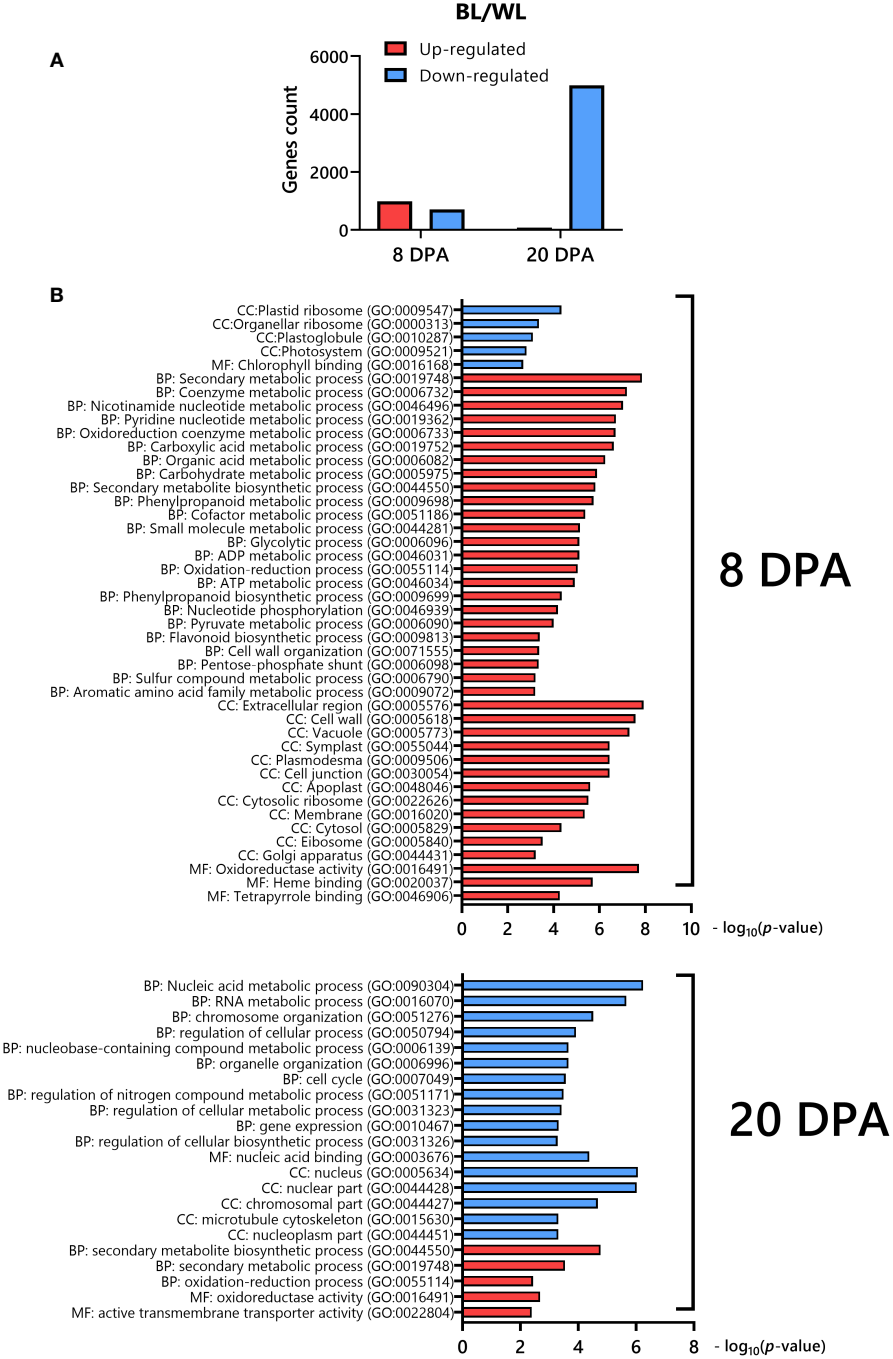
Differentially expressed genes in fibers of NBC compared to its white near-isogenic line. **(A)** Distribution of up-regulated and down-regulated DEGs in NBC. **(B)** Gene ontology (GO) enrichment analysis of DEGs down-regulated (blue) or up-regulated (red) in NBC. The GO terms include those representing cellular components (CC), molecular function (MF), and biological processes (BP).

We analyzed RNAseq data between the NBC and white NILs from developing fibers at 8 and 20 DPA. Two strategically essential time points, including the peak of elongation (8 DPA) and secondary cell wall (SCW) biosynthesis (20 DPA), were selected for RNAseq due to cost efficiency, with two biological replicates minimally required for statistical analysis. A two-way ANOVA determined that 1691 (8 DPA) and 5073 (20 DPA) genes were significantly (FDR < 0.05) differentially expressed (DEGs) between NILs (**Supplementary Data 2**). The count of up-regulated and down-regulated DEGs was 986 and 705, correspondingly, at 8 DPA (**Figure 3A**). However, at 20 DPA, almost all of the DEGs were down-regulated, 4993 genes (**Figure 3A**).

We used an integrated web-based Gene Ontology (GO) analysis toolkit agriGO to analyze the GO enrichment of DEGs (Tian et al., 2017). The GO analysis showed that the up-regulated genes at 8 DPA in the NBC fibers were enriched in secondary metabolism, phenylpropanoid, flavonoid, nucleotide, oxidoreduction, organic acid, carbohydrate, etc. biological processes, cell wall, plasmodesmata, vacuole, apoplast, cytosol, etc. cellular components, and oxidoreductase activity, heme and tetrapyrrole binding molecular functions, whereas down-regulated genes were enriched in plastid ribosome, plastoglobule, photosystem cellular components and chlorophyll binding molecular function (**Figure 3B**). At 20 DPA, the up-regulated DEGs in NBC fibers were enriched in secondary metabolic and oxidation biological processes, oxidoreductase activity, and transmembrane transported activity molecular function. In contrast, down-regulated genes were enriched in nucleic acid, chromosome, cell cycle, etc. biological processes, nucleus, cytoskeleton, nucleoplasm, etc. cellular components, and nucleic acid binding molecular function (**Figure 3B**).

**Figure 4:**
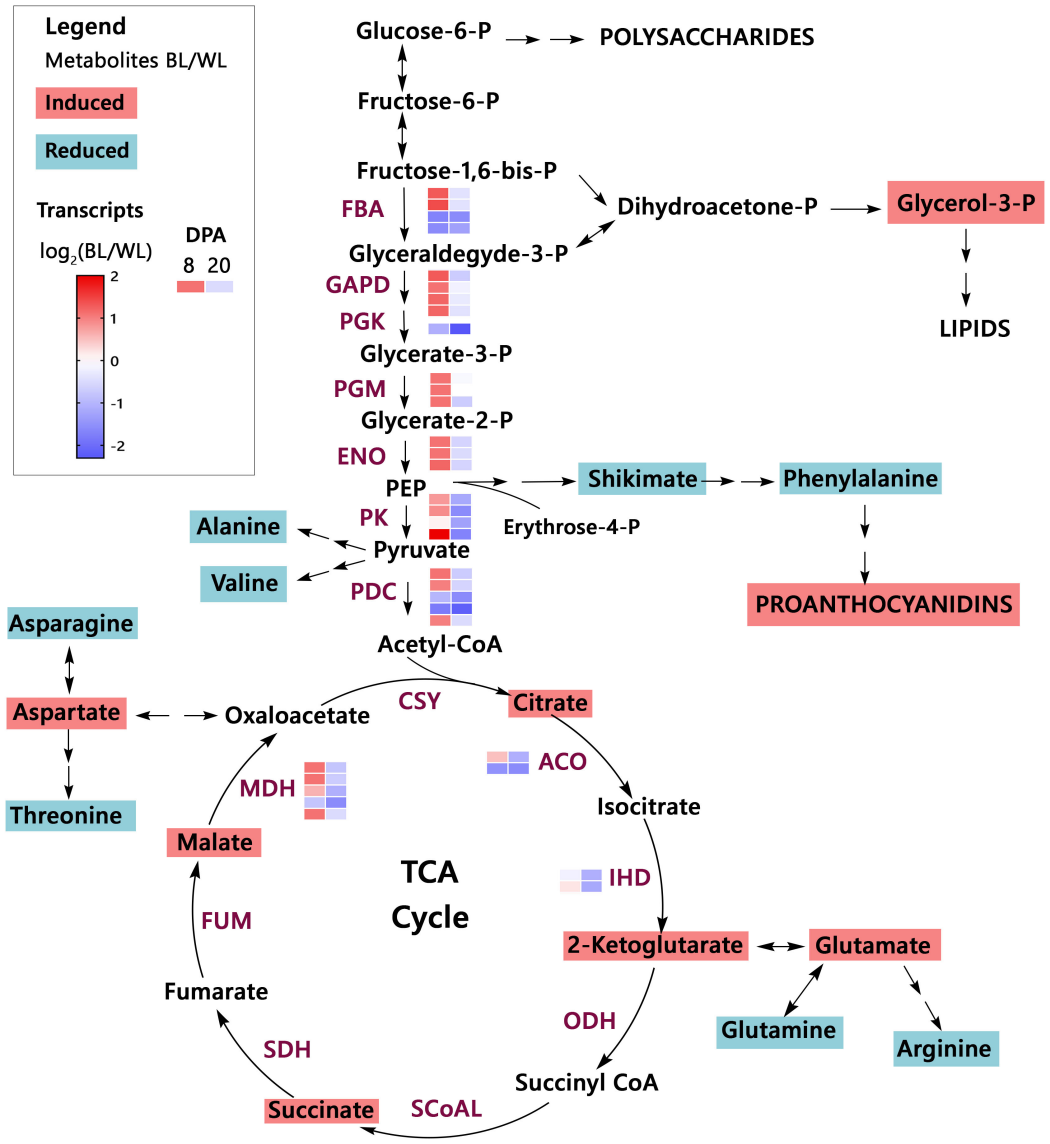
Primary metabolism overview in brown cotton. Metabolites highlighted by magenta color are significantly up-regulated, whereas metabolites highlighted by teal color are down-regulated in MC-BL fibers (**Supplementary Tables 1**, **2**). PEP, phosphoenolpyruvate; TCA cycle, the citric acid cycle. Abbreviations of the enzymes (maroon color): FBA, fructose-bisphosphate aldolase; GAPD, glyceraldehyde 3-phosphate dehydrogenase; PGK, phosphoglycerate kinase; PGM, phosphoglycerate mutase; ENO, enolase; PK, pyruvate kinase; PDC, pyruvate dehydrogenase complex; CSY, citrate synthase; ACO, aconitase; IHD, isocitrate dehydrogenase; ODH, 2-Oxoglutarate Dehydrogenase complex; SCoAL, Succinyl-CoA Ligase; SDH, succinate dehydrogenase; FUM, fumarase; MDH, malate dehydrogenase. The heat map represents the log_2_ ratio of transcripts from fibers (8 and 20 DPA) of brown and white NILs (**Supplementary Table 3**).

Organic acids are synthesized as intermediates of metabolic pathways, representing fixed carbon’s transitory or stored forms. The citric acid cycle (TCA cycle) connects most metabolic pathways and is essential for energy production. **Figure 4** shows changes in the MC-BL fibers in detected metabolites and transcripts of genes encoding enzymes involved in glycolysis and TCA pathways. Most genes from both pathways were up-regulated in MC-BL fibers at 8 DPA and down-regulated at 20 DPA. Genes encoding three enzymes from TCA pathway were significantly differentially expressed between fibers of MC-BL and MC-WL NILs, including aconitase (ACO), isocitrate dehydrogenase (IDH), and malate dehydrogenase (MDH); among them, the MDH was shown to control flux of TCA cycle (Zhang and Fernie, 2018). Glycerol-3-P and detected organic acids from TCA, including citrate, 2-ketoglutarate, succinate, and malate, were significantly (FDR < 0.05) increased in MC-BL compared to MC-WL fibers. In contrast, in MC-BL fibers, shikimate and most amino acids, including phenylalanine, were significantly decreased (**Figure 4**). Thus, the data indicate that significant changes occurred in the primary metabolism of the brown compared to its white NIL.

### 3.3 Proanthocyanidin content in MC-BL and MC-WL fibers

**Figure 5:**
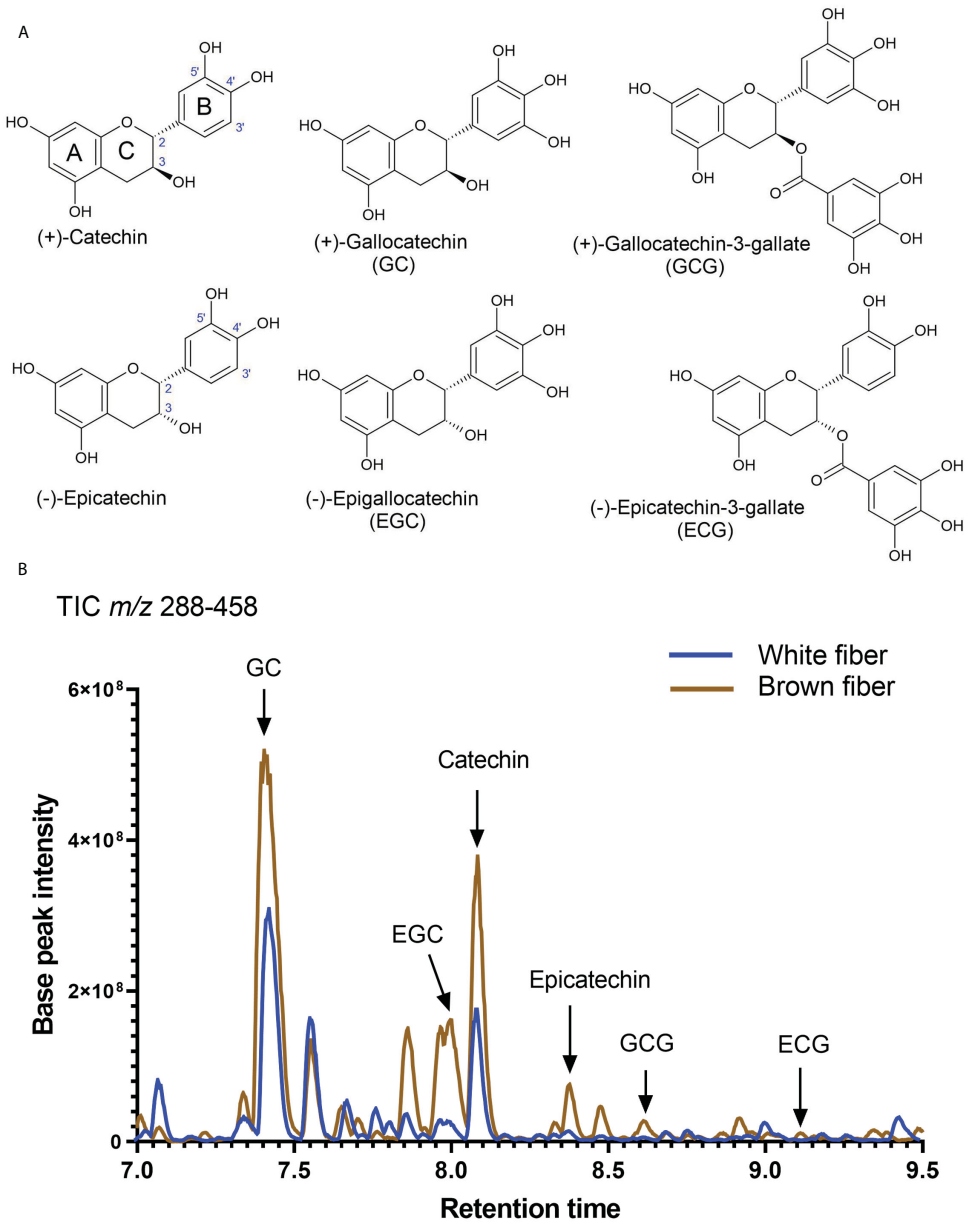
Detection of catechins in brown and white cotton fibers. **(A)** Structures of detected catechins in cotton fibers. **(B)** Total negative-ion UHPLC-MS chromatogram (*m/z* 288-458) from the fiber extract at 20 DPA. A brown line shows chromatograms of compounds detected in the brown fiber sample, whereas the blue line is in the white fiber sample. Arrows indicate the peaks of identified catechins. Abbreviations of catechins are indicated in **(A)**.

Substantial carbon flux from primary metabolism was directed into the PA pathway. Both isoforms of flavan-3-ols, (+)-catechin and (-)-epicatechin, were detected in NBC and white developing and mature fibers. **Figure 5A** shows the structures of catechins and epicatechins with gallate modifications detected in fibers of both NILs. A representative total ion chromatogram of extract from fibers at 20 DPA reveals that detected catechins are present in brown and white fibers; the relative amount of catechin is multiple times greater than epicatechin and their corresponding gallate modifications (**Figure 5B**).

**Figure 6:**
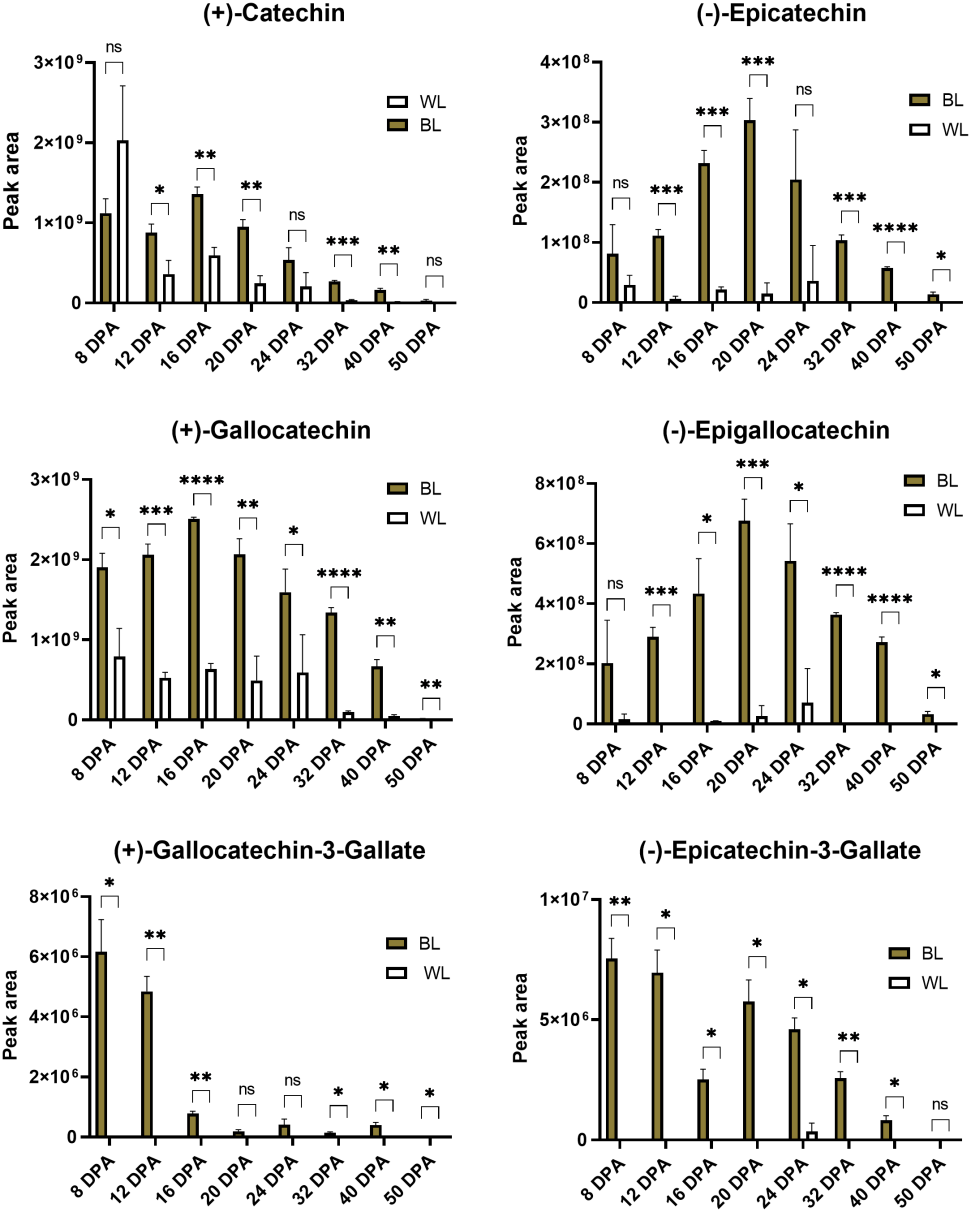
Relative content of catechins based on peak areas in brown and white cotton fibers. Error bars represent standard deviations from three independent biological samples. Asterisks indicate the level of statistical significance determined by multiple comparisons test (Abdi, 2007). *p-value < 0.05, **p-value < 0.01, ***p-value < 0.001, ****p-value < 0.0001, ns, not significant.

**Figure 6** presents relative (based on peak areas) quantifications of catechins, epicatechins, and their gallate modifications in developing and mature fibers of NILs. Catechin and gallocatechin were the most abundant compounds among detected PA subunits. Gallocatechin-3-gallate and Epcatechin-3-gallate accumulated in small amounts, mainly in 8-12 DPA fibers, gradually decreasing during fiber maturation. Catechin, gallocatechin, epicatechin, and epigallocatechin accumulated the most during 16-24 DPA of fiber development in the NBC fibers and substantially less in white fibers. Concentrations of all detected PA subunits were significantly reduced in mature fibers, indicating that they converted into condensed tannins.

### 3.4 GC-MS analyses of fatty acids and long chain alcohols in waxes of mature fibers

**Figure 7:**
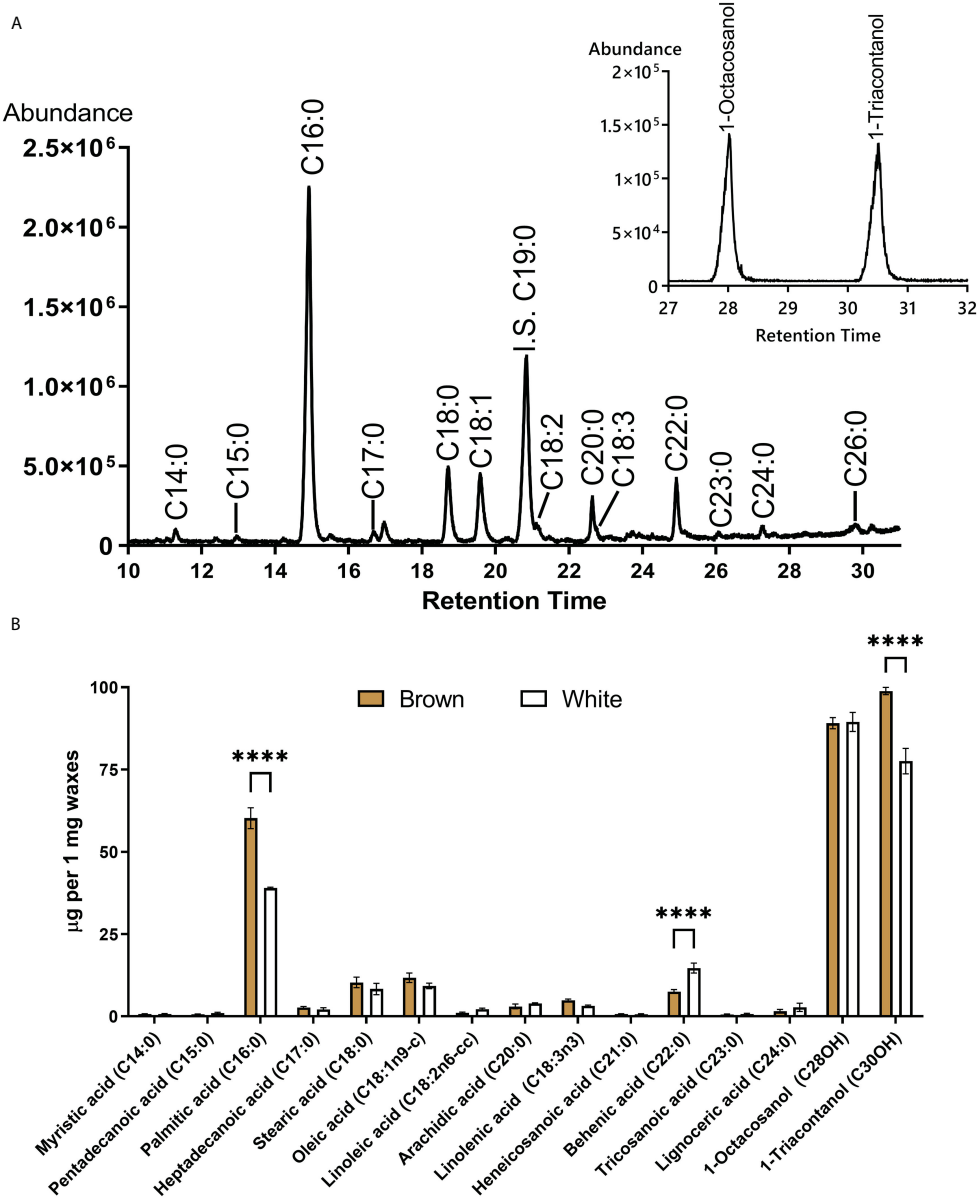
GC-MS of fatty acids and long-chain alcohols from waxes of mature cotton fibers. **(A)** The central panel shows a total ion chromatogram of ester derivatives of fatty acids (from brown fibers). IS an internal standard. Inset represents the base peak chromatogram (m/z 467 + 495) of TMS derivatives of long-chain alcohols. **(B)** Waxes composition from mature fiber of brown and white NILs. Asterisks indicate the level of statistical significance determined by the Mixed model with Bonferroni correction (****p-value < 0.0001).

The composition of waxes extracted from mature fibers was compared between NBC and white NILs. **Figure 7** shows a total ion chromatogram and quantification of ester derivatives of fatty acids extracted from NBC fibers. Palmitic acid (C16:0) and long-chain alcohols, 1-Octcosanol and 1-Triacontanol, were the main components of cotton fiber waxes. Palmitic acid is the building block for many other fatty acids and phospholipids and is widely present in different classes of plant lipids. Palmitic acid and 1-Triacontanol were significantly (FDR < 0.05) higher accumulated in NBC than white fiber waxes (**Figure 7B**). Expression levels of genes involved in biosynthesis fatty acids, including acetyl-CoA carboxylase, acyl-carrier protein, acyl CoA ligase, and 3-ketoacyl-CoA synthase, were significantly up-regulated in NBC developing fibers at 8 DPA but down-regulated at 20 DPA (**Supplementary Table 4**).

### 3.5 EDS analysis of mature fibers

**Figure 8:**
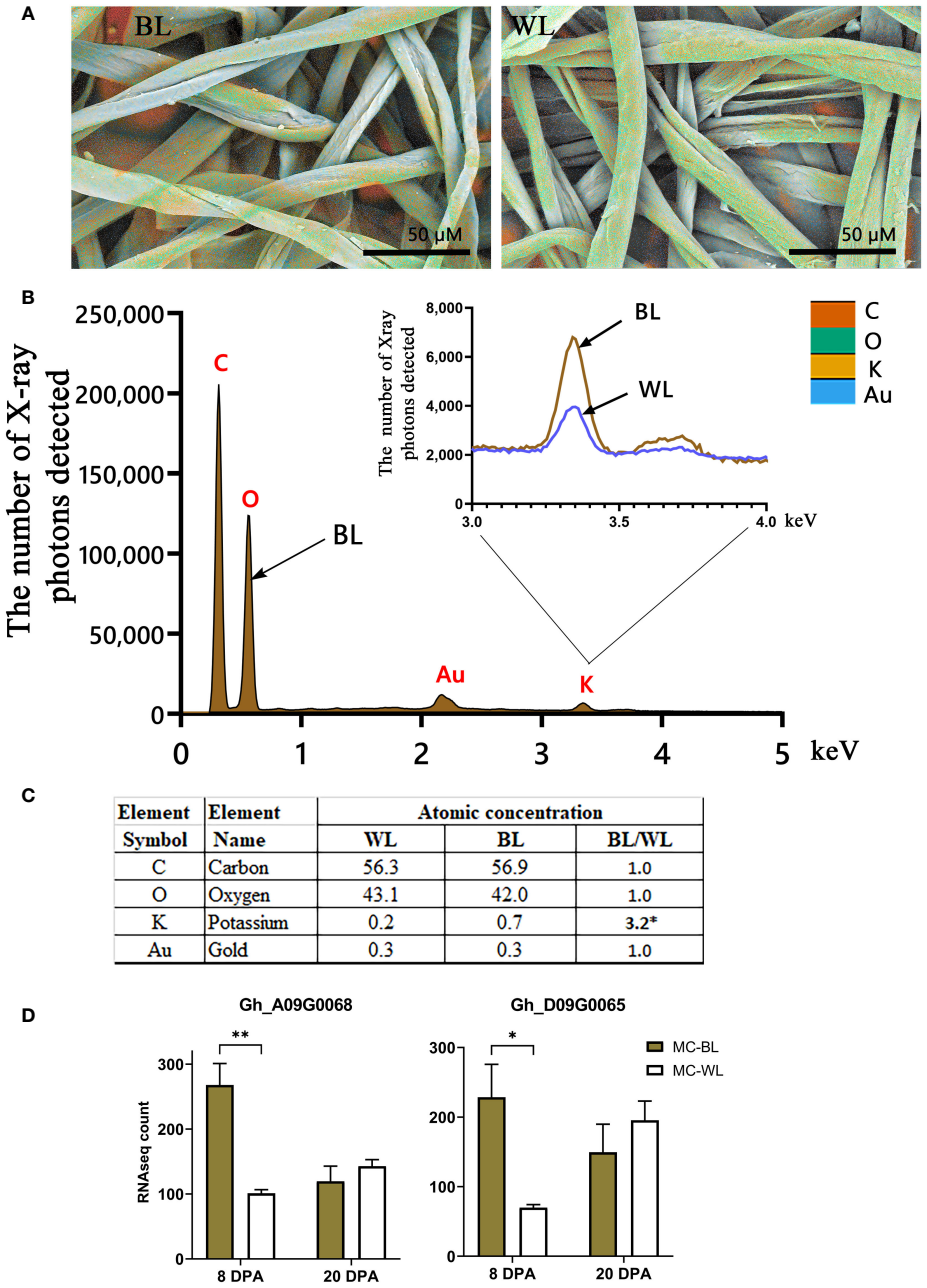
EDS analysis of mature NBC and white fibers. **(A)** EDS color-coded elemental maps embedded in SEM micrographs of mature brown (BL) and white (WL) fibers of NILs. The color code of detected elements is below, on the right. **(B)** EDS spectra for BL mature fibers with inset of magnified potassium peaks from BL and WL fibers. Each element symbol is shown on top of the peak. **(C)** Elemental compositions of BL and WL fibers quantified in atomic percent. The detection limit is approximately 0.1 wt %. The presence of gold resulted from the sputter coating of samples. **(D)** RNAseq expression of two *G hirsutum* genes homologous to *Arabidopsis* TPK3, the two-pore potassium channel. Error bars represent standard deviations from two biological replicates. Asterisks indicate the level of statistical significance determined by multiple comparisons test (Abdi, 2007). *p-value < 0.05, **p-value < 0.01.

It has been previously shown that the presence of various inorganics in raw cotton fibers can alter the thermal properties of cellulose (Nam et al., 2014; Hinchliffe et al., 2015; Nam et al., 2016, 2017). Elemental analysis of the surfaces of NBC and white mature fibers in SEM was conducted using EDS. **Figure 8** shows the EDS results including color-coded elemental maps, EDS spectra, and elemental compositions in atomic percent. Among inorganic elements, only potassium was detected by EDS. The EDS spectra showed that potassium measured in NBC was three times greater than in white fibers (**Figures 8B, C**). To explain the increased potassium level in NBC fibers, we searched transcript data to identify DEGs involved in potassium transport. Two *G. hirsutum* genes homologous to *Arabidopsis*, the two-pore potassium (K+) channel (TPK3), were significantly (FDR < 0.05) up-regulated in NBC developing fiber at 8 DPA (**Figure 8D**). In *Arabidopsis*, the TPK3 regulates the proton gradient in the thylakoid membrane and, therefore, photosynthetic light utilization (Carraretto et al., 2013).

## 4 Discussion

We performed comparative metabolite analyses of developing and mature MC-BL and MC-WL fibers to understand pigmentation processes in NBC and its relationship to FR. The spontaneous mutation occurred in the *Lc1* locus, turning white fiber on the same plant into brown fiber, from which MC-BL and MC-WL NILs originated (Hinchliffe et al., 2016). A 1.4 Mb inversion on chromosome A07 upstream of a TT2 homologous gene (*Gh_A07G2341*) most likely caused up-regulation of the *Gh_A07G2341*, which induced the expression of structural genes in the PA pathway (Hinchliffe et al., 2016).

Previously, independent studies revealed predominance in the accumulation of catechin and gallocatechin (Xiao et al., 2014) or epicatechin and epigallocatechin (Feng et al., 2014) in NBC fibers. Here, we detected both isoforms of flavan-3-ols, (+)-catechin and (-)-epicatechin and their derivatives, in MC-BL and MC-WL fibers; however, catechin and gallocatechin were accumulated multiple times higher than epicatechin and epigallocatechin (**Figures 5**, **6**). What is guiding the PA pathway into the prevalence of *trans*-flavan-3-ols in MC-BL fibers is unclear. Transcript profiling of structural PA genes showed that anthocyanidin synthase (ANS) and anthocyanidin reductase (ANR) were induced in MC-BL much higher than leucoanthocyanidin reductase (LAR) (Hinchliffe et al., 2016; **Figure 5**), expecting higher accumulation of *cis*-flavan-3-ols isoforms. One of the explanations for the higher abundance of *cis*-stereoisomers in MC-BL fibers can be provided by *in vitro* studies that showed ANRs from different species can produce both *cis*- and *trans*-stereoisomers (Xie and Dixon, 2005; Gargouri et al., 2009; Pang et al., 2013; Dixon and Sarnala, 2020); however, this remains to be confirmed *in vivo*.

Induction of the PA pathway in the *Lc1* mutant, MC-BL, leads to condensed tannin accumulation in mature fibers, which reduces fiber length and also affects the primary metabolism during fiber development. At 8 DPA fiber development, up-regulated DEGs involved in metabolic processes such as secondary, nucleotide, oxidoreduction, organic acid, and carbohydrate metabolic processes were significantly enriched in MC-BL (**Figure 3B**). Metabolite analyses revealed that organic acids (TCA cycle) were increased, while most amino acids decreased in MC-BL fibers (**Figure 4**; **Supplementary Tables 1, 2**). TCA metabolic pathway is part of the mitochondrial respiratory apparatus and is responsible for oxidative decarboxylation of organic acids to produce energy in the form of ATP (Millar et al., 2011). TCA connects many metabolic pathways, including carbohydrate, fat, secondary metabolism, and protein biosynthesis. Increasing activity in TCA cycle provides extra energy and carbon sources for PA biosynthesis in MC-BL fibers. We observed a higher accumulation of palmitic acid and 1-triacontanol in MC-BL than in MC-WL, possibly due to increased activity in the TCA cycle.

The source of FR in brown fibers is still a mystery. The condensed tannins are unlikely to be the cause since enhanced FR properties occur in developing fibers before the brown color is detectable. It has been speculated that the unknown FR compound is synthesized in developing fibers and segregated by PA precursors via metal-flavonoid complexes (Hinchliffe et al., 2016). Here, we detected thousands of unknown metabolites accumulated significantly differently between MC-BL and MC-WL fibers; however, which one (or multiple compounds) is responsible for FR in cotton fibers will determine future studies. From another perspective, it has been experimentally demonstrated that different metal ions alter the thermal properties of cotton fibers (Nam et al., 2014; Hinchliffe et al., 2015; Nam et al., 2016, 2017). Among metal ions previously detected, only potassium, the most prevalent inorganic element in cotton fibers, was detected by EDS. Potassium accumulated three times more in brown than white fibers (**Figure 8**). The higher potassium level in brown fibers could be supplied by two up-regulated *G. hirsutum* genes, *Gh_A09G0068* and *Gh_D09G0065*, homologous to *Arabidopsis* TPK3 (the two-pore K+ channel).

## 5 Conclusions

This study found significant changes in thousands of metabolites and transcripts accumulated in MC-WL and MC-BL developing and mature fibers. Organic acids of TCA cycle were induced, while many amino acids were reduced in MC-BL fibers. Both *cis*- and *trans*-stereoisomers of flavan-3-ols were detected in MC-WL and MC-BL fibers. Gene Ontology of up-regulated genes in the MC-BL fibers revealed significant enrichment of genes involved in secondary metabolism, phenylpropanoid, and flavonoid pathways. The gallocatechin and catechin were the main constituents of PA precursors in developing fibers. GC-MS analysis of fatty acids determined that palmitic acid and long-chain alcohols, 1-Octacosanol and 1-Triacontanol, were predominant in cotton fiber waxes. EDS analysis of mature fibers revealed that potassium accumulation was three times greater in MC-BL than in MC-WL fibers.

## Data availability statement

The datasets presented in this study can be found in online repositories. The names of the repository/repositories and accession number(s) can be found below: BioProject, PRJNA326737.

## Author contributions

DH: Conceptualization, Funding acquisition, Investigation, Methodology, Project administration, Resources, Supervision, Writing – review and editing. MN: Data curation, Formal analysis, Software, Validation, Visualization, Writing – original draft, Writing – review and editing. GT: Data curation, Writing – review and editing, Investigation. SN: Data curation, Writing – review and editing, Investigation. SC: Data curation, Writing – review and editing, Investigation. JM: Resources, Writing – review and editing. JJ: Resources, Writing – review and editing.

## Supplementary material

Supplementary Data Sheet 1 A two-way ANOVA analysis of ESI MS Positive and Negative data.

Supplementary Data Sheet 2 A two-way ANOVA analysis of transcript data.
