# Supplementary material for: Multi-omics analysis of pigmentation related to proanthocyanidin biosynthesis in brown cotton (Gossypium hirsutum L.)
Source: Front Plant Sci. 2024 Mar 13;15:1372232. doi: 10.3389/fpls.2024.1372232 (PMC10965557; doi:10.3389/fpls.2024.1372232)
Supplement: Supplementary file 1 [file Table_1.docx]

Supplementary Material

## Supplementary Tables

**Supplementary Table 1.** The down-regulated metabolites in brown fibers. Data show the log_2_ ratios of the fold change of peak area between metabolites identified in brown and white cotton fiber samples. RT, retention time.

| **RT** | **m/z** | **Formula** | **8 dpa** | **12 dpa** | **16 dpa** | **20 dpa** | **24 dpa** | **36 dpa** | **40 dpa** | **50 dpa** | **Name** |
| --- | --- | --- | --- | --- | --- | --- | --- | --- | --- | --- | --- |
| **Amino acids and their derivatives** | | | | | | | | | | | |
| 0.71 | 102.05 | C4H7NO2 | -0.2 | 0.0 | -0.9 | -1.5 | -0.2 | 0.3 | -1.1 | -1.4 | 1-Aminocyclopropane-1-carboxylate |
| 3.36 | 182.08 | C9H11NO3 | -1.3 | -0.6 | -0.1 | -0.8 | -0.4 | 0.1 | -1.1 | -0.3 | 2-Hydroxyphenylalanine |
| 1.58 | 144.08 | C5H11N3O2 | -9.3 | -0.7 | 6.1 | 1.7 | 2.3 | 5.4 | 2.6 | 0.0 | 4-Guanidinobutinoate |
| 0.72 | 132.06 | C5H9NO3 | -1.0 | -1.0 | -2.0 | -1.7 | -0.6 | -1.0 | -2.0 | -2.1 | 4-Hydroxyproline |
| 1.80 | 128.04 | C5H7NO3 | 0.0 | 0.4 | -0.4 | -0.9 | 0.3 | 0.1 | -1.3 | -0.2 | 4-Oxoproline |
| 0.69 | 90.06 | C3H7NO2 | -1.4 | -0.8 | -1.3 | -1.6 | -0.9 | -0.6 | -1.0 | 1.4 | Alanine |
| 0.70 | 131.05 | C4H8N2O3 | -1.6 | -0.8 | -1.7 | -1.7 | -0.1 | -0.1 | -1.5 | -0.1 | Asparagine |
| 7.73 | 188.04 | C10H7NO3 | -11.9 | -8.7 | -6.9 | -3.5 | -3.5 | -1.2 | -0.7 | 2.1 | Kynurenic Acid |
| 0.71 | 173.10 | C6H14N4O2 | -0.3 | 0.0 | -3.8 | -12.3 | 0.7 | 3.4 | -0.8 | -5.4 | L-Arginine |
| 0.71 | 133.06 | C4H8N2O3 | -1.6 | -1.0 | -1.5 | -1.4 | -0.2 | 0.6 | -0.6 | -0.7 | L-Asparagine |
| 0.94 | 162.11 | C7H15NO3 | -1.5 | -2.2 | -2.3 | -2.5 | -1.8 | 0.3 | 0.1 | -2.5 | L-Carnitine |
| 2.55 | 130.09 | C6H13NO2 | -1.9 | -3.0 | -5.4 | -3.0 | -1.4 | -1.2 | -6.1 | -14.2 | Leucine |
| 0.72 | 145.06 | C5H10N2O3 | -3.2 | -2.8 | -4.0 | -3.4 | -0.9 | -0.1 | -1.2 | -2.3 | L-Glutamine |
| 0.70 | 154.06 | C6H9N3O2 | -4.3 | -7.4 | -6.8 | -3.2 | -0.6 | 1.3 | -1.3 | -0.4 | L-Histidine |
| 0.96 | 118.03 | C4H7NOS | -1.7 | -12.3 | -0.2 | 3.3 | -3.5 | 0.6 | -2.6 | 1.1 | L-Homocysteine thiolactone |
| 2.23 | 130.09 | C6H13NO2 | -1.8 | -2.6 | -4.6 | -2.8 | -1.3 | -0.9 | -3.8 | -13.8 | L-Isoleucine |
| 0.79 | 191.10 | C7H14N2O4 | -3.2 | -7.3 | -3.7 | -5.4 | -0.9 | -2.6 | -2.8 | -3.7 | LL-2,6-Diaminoheptanedioate |
| 2.49 | 132.10 | C6H13NO2 | -1.6 | -3.1 | -3.6 | -2.4 | -0.7 | -0.6 | -2.3 | -2.5 | L-Leucine |
| 1.38 | 150.06 | C5H11NO2S | -3.1 | -3.2 | -2.8 | -4.0 | -1.7 | -1.9 | -5.6 | 1.9 | L-Methionine |
| 0.83 | 116.07 | C5H9NO2 | -2.1 | -1.5 | -2.1 | -2.6 | -1.3 | -1.3 | -2.9 | -2.3 | L-Proline |
| 1.11 | 116.07 | C5H11NO2 | -8.2 | -7.8 | -19.9 | -10.2 | -3.6 | -2.2 | -5.1 | -6.1 | L-Valine |
| 2.29 | 154.05 | C5H9NO3 | -1.7 | -1.1 | -2.1 | -1.9 | -1.0 | -1.3 | -2.3 | 0.5 | N-Acetyl-L-alanine |
| 0.85 | 160.06 | C6H11NO4 | -3.9 | 5.2 | -14.8 | -4.1 | -1.4 | 0.2 | -2.2 | -0.8 | N-Methyl-L-glutamate |
| 1.26 | 187.11 | C8H16N2O3 | -16.1 | -15.4 | -8.3 | -12.0 | -2.0 | 0.4 | -0.2 | -8.9 | N-α-Acetyl-L-lysine |
| 0.65 | 133.10 | C5H12N2O2 | -2.8 | 0.6 | 0.9 | 0.1 | -1.3 | 1.3 | 1.4 | -1.4 | Ornithine |
| 6.26 | 166.09 | C9H11NO2 | -3.3 | -8.1 | -3.3 | -2.4 | -0.8 | 0.1 | -1.6 | -0.9 | Phenylalanine |
| 6.27 | 120.08 | C8H10N | -3.4 | -8.2 | -3.3 | -2.4 | -0.8 | 0.1 | -1.6 | -0.9 | Phenylalanine-HCOOH |
| 1.78 | 130.05 | C5H7NO3 | 0.1 | 0.4 | -0.5 | -0.9 | 0.3 | 0.1 | -1.3 | -0.2 | Pyroglutamic Acid |
| 1.15 | 144.10 | C7H13NO2 | -1.9 | -2.0 | -2.2 | -2.7 | -1.4 | -0.1 | 0.2 | -2.5 | Stachydrine |
| 0.68 | 124.01 | C2H7NO3S | -1.7 | -3.6 | -1.4 | -2.1 | -3.6 | -0.8 | 1.6 | -0.1 | Taurine |
| 0.75 | 118.05 | C4H9NO3 | -1.2 | -0.6 | -3.4 | -5.2 | -2.7 | -1.6 | -3.1 | -1.3 | Threonine |
| 7.67 | 203.08 | C11H12N2O2 | -5.5 | -5.4 | -4.0 | -3.1 | -1.7 | -2.8 | -2.2 | 0.1 | Tryptophan |
| 7.67 | 188.07 | C11H10NO2 | -2.2 | -2.4 | -2.1 | -1.9 | -0.1 | -0.1 | -1.4 | 0.0 | Tryptophan-NH3 |
| **RT** | **m/z** | **Formula** | **8 dpa** | **12 dpa** | **16 dpa** | **20 dpa** | **24 dpa** | **36 dpa** | **40 dpa** | **50 dpa** | **Name** |
| **Choline** | | | | | | | | | | | |
| 0.81 | 104.11 | C5H13NO | -1.9 | -1.7 | -1.9 | -1.7 | -1.1 | -0.6 | -0.3 | -0.5 | Choline |
| 0.77 | 258.11 | C8H20NO6P | -4.9 | -4.9 | -5.2 | -4.5 | -1.7 | 0.1 | -0.9 | -2.3 | Glycerophosphocholine |
| 0.74 | 184.07 | C5H14NO4P | -3.1 | -3.7 | -4.0 | -3.4 | 0.0 | 3.3 | 2.6 | -2.0 | Phosphocholine |
| **Miscellaneous** | | | | | | | | | | | |
| 6.13 | 137.05 | C5H4N4O | -6.6 | -1.1 | -3.1 | -1.9 | -2.0 | -0.6 | 2.1 | -0.3 | Allopurinol |
| 0.73 | 171.01 | C3H9O6P | -5.6 | 0.0 | -11.9 | -0.8 | -0.9 | 2.7 | 3.0 | -0.4 | Glycerol 2-phosphate |
| 2.02 | 135.03 | C5H4N4O | -4.1 | -1.2 | 0.3 | -1.8 | -1.6 | 0.1 | 1.2 | -0.2 | Hypoxanthine |
| 9.17 | 174.06 | C10H9NO2 | -3.7 | -2.5 | -12.1 | -2.7 | -0.8 | -0.3 | -0.5 | 3.5 | Indole-3-acetate |
| 1.09 | 131.12 | C6H14N2O | -3.1 | -1.8 | -2.1 | -3.1 | -2.4 | -1.2 | -1.7 | -2.6 | N-Acetylputrescine |
| 6.63 | 218.10 | C9H17NO5 | -1.0 | -1.6 | -2.4 | -1.1 | -0.7 | -0.7 | 0.6 | 0.7 | Pantothenic acid |
| 1.09 | 138.05 | C7H7NO2 | -4.6 | -4.0 | -4.7 | -3.6 | -2.2 | -0.4 | -0.6 | 0.4 | Trigonelline |
| **Organic acids** | | | | | | | | | | | |
| 1.02 | 173.05 | C7H10O5 | -1.7 | -0.4 | -3.0 | -1.2 | -1.5 | -1.8 | 0.9 | -0.3 | Shikimic acid |
| 7.93 | 174.05 | C10H7NO2 | 0.7 | 1.4 | -0.8 | 0.8 | 1.3 | -3.3 | -12.9 | 0.6 | 6-Quinolincarboxylic acid |
| 2.29 | 103.04 | C4H8O3 | 0.4 | 0.4 | -0.3 | -0.1 | 0.0 | -0.2 | -1.1 | -1.9 | α-Hydroxyisobutyric acid |
| **Sugars** | | | | | | | | | | | |
| 0.77 | 256.06 | C8H15NO6 | -0.2 | 1.3 | 0.1 | -0.3 | -0.4 | -1.6 | -1.6 | 0.2 | N-Acetyl-D-mannosamine |
| 0.82 | 220.08 | C8H15NO6 | -1.0 | -0.3 | -1.1 | -2.1 | -1.4 | -1.2 | -1.3 | -0.1 | N-Acetyl-hexosamine |

**Supplementary Table 2.** The up-regulated metabolites in brown fibers. Data show the log_2_ ratios of the fold change of peak area between metabolites identified in brown and white cotton fiber samples. RT, retention time.

| **RT** | **m/z** | **Formula** | **8 dpa** | **12 dpa** | **16 dpa** | **20 dpa** | **24 dpa** | **36 dpa** | **40 dpa** | **50 dpa** | **Name** |
| --- | --- | --- | --- | --- | --- | --- | --- | --- | --- | --- | --- |
| **Amino acids and its derivatives** | | | | | | | | | | | |
| 2.01 | 162.08 | C5H7NO3 | 1.2 | 2.0 | 1.9 | 0.0 | 0.7 | 0.7 | -1.4 | -0.5 | 5-Oxo-L-proline |
| 0.71 | 162.08 | C4H7NO4 | 0.0 | 0.5 | -0.3 | -0.9 | 0.0 | 2.9 | 2.4 | -0.1 | Aspartate |
| 0.76 | 176.04 | C5H9NO4 | 0.2 | 0.3 | 0.3 | -0.9 | -0.2 | 3.1 | 0.3 | -1.2 | L-Glutamic acid |
| 6.87 | 148.06 | C6H11NO3S | -2.6 | 2.1 | -0.9 | 3.1 | 2.3 | 4.1 | 6.3 | 8.5 | N-formyl-L-methionine |
| 0.81 | 132.03 | C6H11NO4 | 1.0 | 1.0 | 0.5 | 1.0 | -0.5 | 1.1 | 2.1 | -0.2 | N-Methyl-L-glutamate |
| **Catechol** | | | | | | | | | | | |
| 6.95 | 152.07 | C8H8O4 | 2.4 | 2.3 | 2.0 | 2.5 | 2.1 | 2.4 | 4.0 | 1.8 | 3,4-Dihydroxyphenylacetate |
| 6.82 | 109.03 | C6H6O2 | 2.0 | 0.6 | -0.1 | -0.1 | 1.6 | 0.7 | 2.2 | 2.9 | Catechol |
| 2.86 | 167.03 | C8H11NO2 | 0.3 | 0.0 | 0.7 | 0.6 | 1.1 | 6.7 | 11.3 | 0.0 | Dopamine |
| **Miscellaneous** | | | | | | | | | | | |
| 0.78 | 305.07 | C2H8NO4P | 0.0 | -0.1 | -0.6 | -1.4 | -0.7 | 3.9 | 2.5 | 0.9 | 2-Aminoethyl dihydrogen phosphate |
| 6.24 | 305.07 | C7H7NO3 | 1.1 | 0.6 | 0.2 | 0.5 | -0.1 | -1.5 | 0.4 | 3.3 | 3-Hydroxyanthranilate |
| 8.23 | 289.07 | C5H6O5 | 3.5 | 3.0 | 1.3 | 0.6 | 1.0 | 1.5 | 1.7 | 1.8 | 4-Hydroxyphenylacetate |
| 0.82 | 303.05 | C4H6N4O3 | 2.7 | 3.2 | 4.5 | 1.5 | 0.6 | 0.2 | -1.4 | 0.0 | Allantoin |
| 8.05 | 289.07 | C9H7NO2 | 0.1 | 0.1 | -1.2 | -0.5 | -0.1 | -2.3 | 1.8 | 4.4 | Dihydroxyquinoine |
| **RT** | **m/z** | **Formula** | **8 dpa** | **12 dpa** | **16 dpa** | **20 dpa** | **24 dpa** | **36 dpa** | **40 dpa** | **50 dpa** | **Name** |
| 6.49 | 457.08 | C6H14O3 | 2.2 | 2.5 | 2.5 | 1.3 | 2.8 | 1.4 | 1.9 | -0.7 | Dipropylene glycol |
| 8.69 | 441.08 | C22H20O10 | 4.7 | 7.6 | 3.0 | 2.9 | 2.6 | 3.6 | 4.9 | 8.7 | Enterocin |
| 6.61 | 140.01 | C10H16O | 0.9 | 0.4 | 0.5 | 0.5 | 0.5 | 0.9 | 1.2 | 4.2 | Pulegone |
| 8.19 | 152.04 | C8H8O3 | 1.4 | 2.1 | 1.2 | -1.1 | 0.2 | 0.3 | 0.5 | 1.5 | Resorcinol monoacetate |
| 6.92 | 162.05 | C10H12N2O | -0.5 | 0.1 | 0.2 | 0.8 | 0.7 | 2.5 | 1.6 | 1.8 | Serotonin |
| 0.68 | 443.10 | C3H9O6P | -1.9 | -0.6 | -1.7 | 0.3 | -0.2 | 4.0 | 4.3 | -2.1 | SN-Glycerol 3-phosphate |
| **Organic acids** | | | | | | | | | | | |
| 1.33 | 153.13 | C5H6N2O4 | -1.0 | 1.6 | 3.2 | -0.3 | 0.3 | 1.7 | 0.2 | -0.5 | (S)-Dihydroorotate |
| 7.80 | 153.05 | C7H6O4 | 1.8 | 2.2 | 1.5 | 2.2 | 1.6 | 1.1 | 2.6 | 1.5 | 2,3-Dihydroxybenzoate |
| 1.52 | 123.04 | C5H8O5 | 2.0 | 1.8 | 1.3 | 0.8 | 1.1 | 1.5 | 0.7 | 0.2 | 2-hydroxyglutarate |
| 1.40 | 151.04 | C5H8O5 | 1.9 | 2.4 | 1.3 | 0.7 | 0.7 | 2.7 | 1.3 | -0.4 | 2-hydroxyglutarate-water |
| 7.38 | 173.02 | C9H9NO4 | -0.5 | -1.9 | -2.4 | 2.5 | 3.5 | 2.5 | 2.1 | 6.2 | 2-Hydroxyhippuric acid |
| 2.23 | 157.04 | C5H8O5 | 2.5 | 2.5 | 1.8 | 0.3 | 0.2 | 0.7 | -0.2 | 0.7 | Citramalate |
| 1.91 | 177.10 | C6H8O7 | 2.6 | 2.8 | 3.5 | 2.1 | 1.8 | 3.8 | 2.3 | 0.4 | Citrate |
| 8.09 | 157.08 | C6H14O3S | 1.0 | 3.0 | 2.7 | 0.8 | 1.0 | 0.0 | 1.8 | 3.7 | Hexanesulfonic acid |
| 1.15 | 157.03 | C6H8O7 | 1.8 | 1.7 | 1.6 | 1.7 | 1.6 | 3.1 | 2.2 | 0.7 | Isocitric acid |
| 1.07 | 145.01 | C4H6O5 | 1.0 | 1.1 | 1.0 | 0.3 | 0.3 | 0.4 | -0.1 | -0.3 | Malate |
| 1.08 | 285.04 | C4H6O5 | 1.2 | 1.3 | 1.5 | 0.7 | 0.4 | 0.5 | -0.2 | -0.4 | (R)-Malate |
| 1.44 | 141.02 | C6H9NO5 | 1.2 | 1.1 | 1.1 | -0.1 | 0.4 | 1.0 | -0.2 | 1.9 | N-Acetyl-L-aspartic acid |
| 7.95 | 194.05 | C9H8O3 | -0.2 | 1.0 | 0.2 | 1.3 | 1.5 | 1.4 | 2.6 | 3.4 | Phenylpyruvate |
| 0.83 | 163.04 | C7H12O6 | -0.2 | -0.1 | 0.5 | -0.1 | 0.7 | 1.0 | 1.3 | 1.1 | Quinate |
| 2.25 | 157.01 | C4H6O4 | -0.2 | -0.8 | -0.5 | -0.8 | -0.7 | 0.1 | 1.1 | 0.3 | Succinate |
| 0.86 | 147.03 | C4H6O6 | 4.1 | 3.5 | 2.9 | 2.1 | 1.5 | 0.2 | 0.4 | -0.7 | Tartaric acid |
| 0.99 | 133.01 | C5H6O5 | 0.9 | 1.5 | 0.9 | 0.7 | 0.6 | -0.5 | -1.2 | 0.1 | α-Ketoglutaric acid |
| **Phenylpropanoids** | | | | | | | | | | | |
| 7.60 | 191.06 | C7H6O4 | 1.4 | 0.8 | 1.5 | 1.8 | 1.3 | 0.4 | 2.6 | 2.0 | 2,5-Dihydroxybenzoate |
| 6.69 | 165.06 | C7H6O4 | 3.0 | 3.2 | 1.5 | 1.1 | 0.8 | 1.1 | 2.9 | 4.6 | 3,4-Dihydrohybenzoate |
| 8.26 | 176.05 | C9H8O3 | 0.8 | 0.1 | -0.3 | 1.4 | 1.6 | 1.8 | 2.4 | 4.2 | 4-Coumarate |
| 8.01 | 149.01 | C7H6O2 | 1.1 | 2.7 | 1.2 | 1.9 | 2.3 | 3.4 | 2.3 | -0.1 | 4-Hydroxybenzaldehyde |
| 7.42 | 147.03 | C7H6O3 | 1.9 | 2.7 | 2.3 | 1.4 | 1.5 | 0.5 | 1.1 | 1.1 | 4-Hydroxybenzoate |
| 9.34 | 131.03 | C9H8O4 | 24.0 | 26.2 | 18.8 | 3.7 | 3.2 | 3.9 | 3.7 | 0.6 | Caffeic Acid |
| 8.09 | 191.02 | C15H14O6 | -0.2 | 1.4 | 1.2 | 2.0 | 1.7 | 3.0 | 3.6 | 4.7 | Catechin |
| 8.33 | 191.02 | C15H14O6 | 0.4 | 4.5 | 3.4 | 5.0 | 4.5 | 6.4 | 5.9 | 8.5 | Epicatechin |
| 9.25 | 195.06 | C22H18O10 | 19.1 | 22.6 | 17.2 | 9.8 | 7.6 | 9.9 | 12.2 | 11.3 | Epicatechin Gallate |
| 7.94 | 137.02 | C15H14O7 | 4.8 | 7.0 | 5.4 | 5.8 | 4.7 | 7.8 | 7.5 | 6.2 | Epigallocatechin |
| 8.48 | 315.11 | C10H10O4 | -1.8 | -0.2 | -1.3 | -0.2 | 1.6 | 3.0 | 3.1 | 2.0 | Ferulate |
| 7.43 | 179.03 | C15H14O7 | 1.4 | 2.0 | 2.0 | 2.2 | 1.7 | 3.8 | 3.8 | 2.0 | Gallocatechin |
| 8.52 | 193.05 | C22H18O11 | 18.8 | 22.1 | 11.7 | 4.3 | 4.9 | 9.6 | 14.7 | 13.9 | Gallocatechin Gallate |
| 11.31 | 155.03 | C15H10O6 | -2.3 | 1.5 | -2.2 | 1.3 | 3.6 | 5.7 | 4.8 | 7.1 | Kaempferol |
| 9.97 | 163.04 | C10H10O4 | -0.3 | 2.8 | 1.6 | 2.2 | 1.8 | 2.2 | 2.9 | 3.2 | Monoethyl phthalate |
| 9.40 | 153.02 | C7H6O3 | 2.1 | 2.8 | 1.1 | 1.2 | 0.6 | -0.3 | 0.8 | 0.5 | Salicilate |
| 9.30 | 153.02 | C15H12O7 | 1.1 | 3.8 | 2.8 | 3.6 | 3.4 | 4.7 | 8.1 | 7.7 | Taxifolin |
| 7.86 | 137.02 | C14H20O8 | -0.6 | -0.8 | -0.9 | -1.0 | -1.0 | -0.4 | 2.4 | 4.2 | Vanilloloside |
| **Sugars** | | | | | | | | | | | |
| 0.74 | 149.05 | C6H13O10P | 12.6 | 16.6 | 11.8 | 1.8 | 0.0 | 3.2 | 5.7 | 1.5 | 6-Phosphogluconic acid |
| 0.87 | 260.05 | C18H32O16 | -0.9 | -0.7 | -0.6 | -0.2 | 0.2 | 1.2 | 4.7 | -1.3 | D-(+)-Raffinose |
| 0.80 | 168.99 | C6H10O7 | 0.7 | 1.2 | 1.0 | -0.5 | -0.1 | 0.5 | 1.2 | 1.2 | D-Glucoronic acid |
| 0.76 | 173.04 | C6H14NO8P | 3.1 | 1.7 | 1.0 | 1.0 | 1.1 | -0.2 | -1.7 | 0.4 | D-Glucosamine 6-P |
| 0.76 | 180.09 | C3H7O6P | 0.6 | 0.3 | -0.9 | 1.2 | 0.2 | 5.4 | 1.4 | 0.1 | Dihydroxyacetone P |
| 0.77 | 215.03 | C5H10O5 | -0.9 | -0.3 | 0.1 | -0.3 | 3.5 | 1.7 | 0.0 | 0.3 | D-Ribose |
| 0.74 | 89.02 | C6H10O8 | 0.2 | 0.9 | 0.7 | 0.3 | 0.1 | 1.8 | 1.7 | -0.8 | D-Saccharic acid |
| 0.73 | 209.03 | C6H12O7 | 0.7 | 1.3 | 1.2 | 0.3 | -0.2 | 0.5 | 2.1 | 0.6 | Gluconic acid |
| 0.75 | 275.02 | C6H13NO5 | 0.1 | 0.2 | 0.2 | 0.4 | 0.0 | 0.3 | 2.1 | 0.9 | Glucosamine/mannosamine |
| 0.74 | 193.04 | C6H12O6 | -0.1 | -0.2 | -0.1 | -0.1 | -0.2 | 0.0 | 1.5 | 0.5 | Glucose/fructose |
| 0.77 | 195.05 | C3H6O3 | -0.3 | -0.4 | -0.3 | -0.1 | -0.7 | 0.3 | 2.0 | -0.5 | Glyceraldehyde |
| **RT** | **m/z** | **Formula** | **8 dpa** | **12 dpa** | **16 dpa** | **20 dpa** | **24 dpa** | **36 dpa** | **40 dpa** | **50 dpa** | **Name** |
| 0.82 | 193.07 | C3H6O4 | 1.7 | 2.2 | 1.3 | 0.6 | 0.5 | 0.6 | 1.2 | -0.5 | Glyceric acid |
| 1.03 | 105.02 | C7H14O6 | 0.9 | 1.4 | 0.9 | 0.5 | 0.6 | 1.7 | 1.3 | 0.3 | Methyl β-D-galactoside |
| **Vitamins** | | | | | | | | | | | |
| 0.89 | 121.04 | C6H8O6 | 0.7 | 1.4 | 1.2 | 0.8 | 0.5 | 1.4 | 1.2 | -1.0 | Ascorbic acid |
| 2.86 | 168.07 | C8H11NO3 | -0.7 | -0.6 | -0.1 | -0.3 | 0.7 | 2.5 | 2.0 | 5.4 | Pyridoxine |
| 1.09 | 173.01 | C12H17N4O4PS | 1.9 | 2.4 | 0.5 | 0.9 | 0.6 | 1.6 | 1.3 | 0.1 | Thiamine monophosphate |

**Supplementary Table 3**. Glycolysis and TCA cycle metabolism DEGs. Numbers represent fold change of transcript level between MC-BL and MC-WL.

| **Enzyme** | **Arabidopsis** | ***G. hirsutum*** | **8 DPA** | **20 DPA** |
| --- | --- | --- | --- | --- |
| **Glycolysis** | | | | |
| Fructose-bisphosphate aldolase | AT2G36460 | Gh_A01G1785 | 1.3 | -0.4 |
|  | AT3G52930 | Gh_A04G0547 | 1.4 | -0.4 |
|  | AT4G38970 | Gh_D13G0294 | -1.7 | -1.6 |
|  |  | Gh_D13G0295 | -1.6 | -1.3 |
| Glyceraldehyde 3-phosphate dehydrogenase | AT1G16300 | Gh_A01G0785 | 1.3 | -0.7 |
|  |  | Gh_D11G2927 | 1.1 | -0.2 |
|  | AT3G04120 | Gh_D04G0765 | 1.2 | -0.3 |
|  |  | Gh_A05G2976 | 1.2 | -0.4 |
| Phosphoglycerate kinase | AT1G79550 | Gh_A06G0769 | -1.1 | -2.3 |
| Phosphoglycerate mutase | AT1G22170 | Gh_D06G0340 | 1.1 | -0.1 |
|  |  | Gh_A06G0310 | 1.1 | 0.0 |
|  | AT1G09780 | Gh_D02G1082 | 1.1 | -0.7 |
| Enolase | AT1G74030 | Gh_A03G0649 | 1.1 | -0.6 |
|  | AT2G36530 | Gh_D01G2033 | 1.1 | -0.5 |
|  |  | Gh_D09G2437 | 1.2 | -0.6 |
| Pyruvate kinase | AT3G52990 | Gh_A09G1335 | 0.8 | -1.2 |
|  |  | Gh_D09G2442 | 0.9 | -1.6 |
|  | AT3G52990 | Gh_A10G1783 | 0.1 | -1.3 |
|  | AT5G08570 | Gh_A13G0547 | 2.0 | -1.7 |
| **The citric acid cycle** | | | | |
| Pyruvate Dehydrogenase Complex | AT2G34590 | Gh_A05G1243 | 1.1 | -0.7 |
|  |  | Gh_D05G1406 | 1.0 | -0.6 |
|  | AT5G50850 | Gh_A12G1299 | -1.0 | -1.6 |
|  |  | Gh_D12G1421 | -1.8 | -2.2 |
|  | AT3G13930 | Gh_D06G2064 | 1.0 | -0.5 |
| Aconitase | AT2G05710 | Gh_D05G3734 | 0.5 | -1.1 |
|  | AT4G35830 | Gh_A02G1668 | -1.5 | -1.6 |
| Isocitrate Dehydrogenase | AT1G54340 | Gh_D13G1335 | -0.2 | -1.1 |
|  |  | Gh_A13G1069 | 0.2 | -1.2 |
| Malate Dehydrogenase  Malate Dehydrogenase | AT5G43330 | Gh_D02G0438 | 1.1 | -0.8 |
|  |  | Gh_A10G2294 | 1.1 | -0.7 |
|  | AT1G53240 | Gh_A04G0320 | 0.6 | -1.1 |
|  | AT3G15020 | Gh_A07G1844 | -0.8 | -1.6 |
|  | AT3G47520 | Gh_D07G0663 | 1.1 | -0.5 |

**Supplementary Table 4.** Fatty acids synthesis. Numbers represent fold change of transcript level between MC-BL and MC-WL.

| **Enzyme** | **Arabidopsis** | ***G. hirsutum*** | **8 DPA** | **20 DPA** |
| --- | --- | --- | --- | --- |
| Acetyl-CoA carboxylase | AT1G36160 | Gh_A01G1574 | 1.4 | -0.7 |
|  |  | Gh_D01G1885 | 1.6 | -0.3 |
|  |  | Gh_Sca054591G01 | 2.1 | -1.2 |
| Acyl-carrier protein (ACP) | AT3G15690 | Gh_D10G0709 | -1.2 | -1.1 |
| Acyl-carrier protein (ACP) | AT2G44620 | Gh_A08G0514 | 1.2 | -1.0 |
| Ketoacyl ACP synthase | AT1G62640 | Gh_A09G1749 | 0.2 | -1.1 |
|  |  | Gh_A11G0242 | -0.4 | -2.1 |
|  |  | Gh_D11G0257 | 0.2 | -1.8 |
|  | AT1G74960 | Gh_D08G2566 | 0.2 | -1.0 |
|  | AT2G04540 | Gh_A11G1407 | -0.6 | -1.1 |
| Enoyl ACP reductase | AT2G05990 | Gh_A08G0113 | 0.7 | -1.0 |
|  |  | Gh_A13G1854 | -0.1 | -1.6 |
| ACP thioesterase | AT1G08510 | Gh_D13G2098 | 0.2 | -1.1 |
|  |  | Gh_A13G1750 | 0.2 | -1.5 |
|  | AT3G25110 | Gh_D08G2090 | 0.2 | -1.0 |
| Acyl CoA ligase | AT1G55320 | Gh_D13G2223 | 1.2 | -0.4 |
|  |  | Gh_A13G2347 | 1.1 | -0.6 |
|  | AT2G17650 | Gh_A02G1693 | -1.3 | -1.1 |
|  | AT3G16170 | Gh_D10G0649 | 0.5 | -1.2 |
|  | AT3G23790 | Gh_D10G2438 | -1.5 | -1.0 |
|  | AT3G48990 | Gh_A08G2389 | -1.4 | -1.8 |
|  | AT5G16340 | Gh_D09G1937 | 1.1 | 0.2 |
|  | AT5G35930 | Gh_A13G2165 | -1.6 | -1.2 |
|  |  | Gh_D13G1022 | -1.2 | -1.3 |
| Long chain fatty acid CoA ligase | AT1G77590 | Gh_A06G0374 | 0.5 | -1.2 |
|  | AT2G47240 | Gh_A07G1181 | 0.3 | -1.6 |
|  |  | Gh_D07G1281 | -0.2 | -2.6 |
| 3-Ketoacyl-CoA synthase | AT1G01120 | Gh_D12G1163 | 1.0 | 0.8 |
|  |  | Gh_A12G1044 | 1.0 | 0.7 |
|  |  | Gh_D07G1331 | -1.1 | 1.2 |
|  | AT5G04530 | Gh_A06G1558 | 1.0 | -0.7 |
|  |  | Gh_Sca144453G01 | 1.3 | -0.4 |
